# Atherosclerotic burden and cerebral small vessel disease: exploring the link through microvascular aging and cerebral microhemorrhages

**DOI:** 10.1007/s11357-024-01139-7

**Published:** 2024-04-19

**Authors:** Anna Csiszar, Anna Ungvari, Roland Patai, Rafal Gulej, Andriy Yabluchanskiy, Zoltan Benyo, Illes Kovacs, Peter Sotonyi, Angelia C. Kirkpartrick, Calin I. Prodan, Eric M. Liotta, Xin A. Zhang, Peter Toth, Stefano Tarantini, Farzaneh A. Sorond, Zoltan Ungvari

**Affiliations:** 1 Vascular Cognitive Impairment, Neurodegeneration and Healthy Brain Aging Program, Department of Neurosurgery, University of Oklahoma Health Sciences Center, Oklahoma City, OK USA; 2 Stephenson Cancer Center, University of Oklahoma, Oklahoma City, OK USA; 3 Oklahoma Center for Geroscience and Healthy Brain Aging, University of Oklahoma Health Sciences Center, Oklahoma City, OK USA; 4 Department of Public Health, Semmelweis University, Semmelweis University, Budapest, Hungary; 5 Department of Health Promotion Sciences, College of Public Health, University of Oklahoma Health Sciences Center, Oklahoma City, OK USA; 6 International Training Program in Geroscience, Doctoral College/Department of Public Health, Semmelweis University, Budapest, Hungary; 7 Institute of Translational Medicine, Semmelweis University, 1094 Budapest, Hungary; 8 Cerebrovascular and Neurocognitive Disorders Research Group, HUN-REN, Semmelweis University, 1094 Budapest, Hungary; 9 Department of Ophthalmology, Semmelweis University, 1085 Budapest, Hungary; 10 Department of Ophthalmology, Weill Cornell Medical College, New York, NY 10021 USA; 11 Department of Vascular and Endovascular Surgery, Heart and Vascular Centre, Semmelweis University, 1122 Budapest, Hungary; 12 Veterans Affairs Medical Center, Oklahoma City, OK USA; 13 Department of Medicine, University of Oklahoma Health Sciences Center, Oklahoma City, OK USA; 14 Department of Neurology, University of Oklahoma Health Sciences Center, Oklahoma City, OK USA; 15 Department of Neurology, Division of Stroke and Neurocritical Care, Northwestern University Feinberg School of Medicine, Chicago, IL USA; 16 Department of Physiology, University of Oklahoma Health Science Center, Oklahoma City, OK USA; 17 Department of Neurosurgery, Medical School, University of Pecs, Pecs, Hungary; 18 Neurotrauma Research Group, Szentagothai Research Centre, University of Pecs, Pecs, Hungary; 19 ELKH-PTE Clinical Neuroscience MR Research Group, University of Pecs, Pecs, Hungary

**Keywords:** Atherosclerosis, Arteriosclerosis, Aging, Peripheral artery disease, White matter injury, White matter hyperintensities, Leukoaraiosis, Blood–brain barrier, Microbleed, Stroke, Vascular dementia

## Abstract

Cerebral microhemorrhages (CMHs, also known as cerebral microbleeds) are a critical but frequently underestimated aspect of cerebral small vessel disease (CSVD), bearing substantial clinical consequences. Detectable through sensitive neuroimaging techniques, CMHs reveal an extensive pathological landscape. They are prevalent in the aging population, with multiple CMHs often being observed in a given individual. CMHs are closely associated with accelerated cognitive decline and are increasingly recognized as key contributors to the pathogenesis of vascular cognitive impairment and dementia (VCID) and Alzheimer’s disease (AD). This review paper delves into the hypothesis that atherosclerosis, a prevalent age-related large vessel disease, extends its pathological influence into the cerebral microcirculation, thereby contributing to the development and progression of CSVD, with a specific focus on CMHs. We explore the concept of vascular aging as a continuum, bridging macrovascular pathologies like atherosclerosis with microvascular abnormalities characteristic of CSVD. We posit that the same risk factors precipitating accelerated aging in large vessels (i.e., atherogenesis), primarily through oxidative stress and inflammatory pathways, similarly instigate accelerated microvascular aging. Accelerated microvascular aging leads to increased microvascular fragility, which in turn predisposes to the formation of CMHs. The presence of hypertension and amyloid pathology further intensifies this process. We comprehensively overview the current body of evidence supporting this interconnected vascular hypothesis. Our review includes an examination of epidemiological data, which provides insights into the prevalence and impact of CMHs in the context of atherosclerosis and CSVD. Furthermore, we explore the shared mechanisms between large vessel aging, atherogenesis, microvascular aging, and CSVD, particularly focusing on how these intertwined processes contribute to the genesis of CMHs. By highlighting the role of vascular aging in the pathophysiology of CMHs, this review seeks to enhance the understanding of CSVD and its links to systemic vascular disorders. Our aim is to provide insights that could inform future therapeutic approaches and research directions in the realm of neurovascular health.

## Introduction

Cerebral small vessel disease (CSVD) stands as a pivotal yet often underappreciated component of age-related neurovascular disorders [1–5]. It encompasses a range of pathologies impacting the small arteries, arterioles, capillaries, and postcapillary venules within the brain, playing a major role in stroke, dementia, and age-related cognitive decline. Clinically significant, CSVD accounts for approximately 25% of ischemic strokes and a substantial 85% of intracerebral hemorrhages [6]. It represents as a principal factor in the onset and progression of vascular cognitive impairment and dementia (VCID). Moreover, CSVD notably contributes to the pathogenesis of a majority of dementias, including those classified under the Alzheimer’s disease spectrum [7]. This highlights its pivotal role in both vascular and neurodegenerative brain disorders.

Neuropathologically, CSVD encompasses a range of pathologies affecting perforating arteries, arterioles, capillaries, and veins within the brain parenchyma and the leptomeningeal vessels. Histologically, CSVD is marked by conditions such as arteriolosclerosis, lipohyalinosis, fibrinoid necrosis, and cerebral amyloid angiopathy (CAA), among others [8]. Large autopsy series have demonstrated a high prevalence of these histopathological signs in older adults, indicating a strong association with VCID. For instance, autopsy data from 1474 older participants in the Rush Alzheimer’s Disease Center studies revealed that 80% of the decedents had neuropathological signs of cerebrovascular disease [9]. In the Religious Orders Study and Memory and Aging Project (ROSMAP) cohort, arteriolosclerosis was identified in 33.5% of cases, a prevalence comparable to that of large vessel atherosclerosis, which was found in 33.1% of cases [9]. Two-thirds of these cases exhibited mixed cerebrovascular pathologies, displaying a co-occurrence of both large vessel and microvascular pathologies [9]. Particularly striking is the observation that the patient subgroup with concurrent atherosclerosis and arteriosclerosis experienced the most rapid cognitive decline across all domains, compared to subgroups with any single type of cerebrovascular pathology [9]. These data underscore the widespread nature of CSVD in the aging population and the potential impact of combined vascular pathologies on cognitive health.

Despite their significance, these small vessel pathologies remain challenging to directly observe through standard clinical neuroimaging. Advanced techniques like 7 T MRI have suggested potential visualization, but typically, CSVD is identified through neuroimaging and pathological features revealing parenchymal damage linked to clinical symptoms. Imaging signs of CSVD, such as lacunar infarcts, microinfarcts, enlarged perivascular spaces (PVS), diffuse white matter lesions, seen as white matter hyperintensities (WMHs) on T2-weighted MRI images, and cerebral microhemorrhages (CMHs), are vital for in vivo visualization, with MRI being the most effective tool [1, 3, 10–12]. The prevalence of these imaging signs is alarmingly high in the aging populations of the Western world, and their association with cognitive impairment, VCID, and AD is well-established [1, 11, 12]. Imaging-histological correlations in CSVD are evolving, with some lesions like CMHs, lacunar infarcts, PVS, and small infarcts being identifiable both by MRI and histologically [13]. However, others, such as early-stage WMHs, are more difficult to assess by histology than MRI [13].

Central to CSVD is the pathological manifestation of CMHs, also known as cerebral microbleeds [14]. These small, dot-like lesions, detectable through advanced neuroimaging, exemplify microvascular damage and hold significant clinical relevance [14]. CMHs are associated with an increased risk of stroke and cognitive impairment, and are prominent in VCID and AD [14]. Their prevalence in the aging population points to advanced microvascular pathology and a heightened risk of neurodegenerative processes [14]. The study of CMHs is of paramount importance in understanding and managing CSVD, particularly given their potential as preventable consequences of this disease. Recent animal studies have shed light on various aspects of CMH pathogenesis, suggesting that targeting the underlying mechanisms could lead to clinically translatable preventive strategies [14–22]. CMHs, often found in conjunction with other CSVD markers, not only reflect the current state of microvascular health but also provide critical insights into the progression and potential mitigation of age-related neurovascular disorders. Their role in cognitive decline and associations with large vessel pathologies underscore their significance in both clinical research and patient care. By focusing on CMHs, we can better comprehend and potentially curb the advancement of CSVD, ultimately improving outcomes for aging populations at risk of neurovascular and cognitive impairments.

Aging and hypertension are recognized as primary risk factors for CMHs [14]. Recent preclinical studies have begun to unravel how fundamental aging mechanisms contribute to the pathogenesis of CMHs [14–22]. These studies reveal that aging processes, including cellular senescence, oxidative stress, and endothelial dysfunction, lead to increased microvascular fragility [20, 22]. This fragility predisposes the cerebral microvasculature to high pressure/increased wall tension-induced ruptures, culminating in the formation of CMHs. These insights are crucial in understanding the molecular and cellular pathways that drive the development of CMHs in aging populations.

However, a comprehensive understanding of the origins of CSVD and CMHs also necessitates an exploration of large vessel pathologies associated with aging, particularly atherosclerosis [14, 23, 24]. Atherosclerosis, an age-related inflammatory vascular disease, is marked by the thickening and hardening of arterial walls due to plaque accumulation. Increasingly, evidence suggests that the pathological process underlying atherogenesis extends beyond large vessels to influence microvascular health, potentially contributing to CSVD and the formation of CMHs. This evolving perspective underscores the interconnectedness of macrovascular and microvascular pathologies in the aging vasculature, highlighting the need for integrated approaches in research and clinical interventions.

This review paper seeks to bridge the knowledge gap between macrovascular pathologies like atherosclerosis and their impact on microvascular health, especially regarding CSVD and CMHs. We hypothesize that mechanisms driving atherosclerosis and large vessel aging are also fundamental to microvascular changes leading to CSVD. Our goal is to elucidate the interconnected nature of these vascular processes and their cumulative impact on cerebral health. We review existing epidemiological evidence, delve into shared pathophysiological mechanisms, and highlight the clinical significance of this vascular continuum. Our objective is to provide a comprehensive understanding of how atherosclerosis as an age-related large vessel disease intertwines with microvascular aging and pathology, shaping the landscape of CSVD and its manifestations, including CMHs.

## Epidemiology and clinical impact of CMHs

As emerging research sheds light on the epidemiology and clinical impact of CMHs, their significance in both aging populations and individuals with various vascular risk factors becomes increasingly apparent. This section delves into the detection methods for CMHs, explores their prevalence and patterns in different demographic groups, and examines their association with cognitive decline, vascular cognitive impairment, dementia, and Alzheimer’s disease. Furthermore, we will explore the intricate connections between CMHs and various manifestations of atherosclerotic vascular diseases, including carotid stenosis, myocardial infarction, ischemic stroke, and peripheral arterial disease. Through comprehensive reviews of recent studies and analyses, we aim to provide a thorough understanding of the implications of CMHs in the broader context of vascular health and cerebrovascular pathology.

### Detection of CMHs through advanced imaging techniques

CMHs are effectively detected using advanced neuroimaging MRI techniques, particularly T2*-weighted imaging [10, 14]. Both gradient echo (GRE) and susceptibility-weighted imaging (SWI) are sensitive to blood and iron products and are particularly useful for detecting CMHs. CMHs manifest as small, round, or ovoid hypointense (dark) lesions on these scans. CMHs are generally defined as lesions measuring up to 10 mm in diameter. Of the two neuroimaging methods, SWI is noted for its higher sensitivity, capable of identifying smaller and more subtle CMHs that might be missed on GRE imaging.

### Prevalence of CMHs in the aging population

The prevalence of CMHs significantly increases with age [25], becoming more common in older adults and often co-occurring with other imaging signs of CSVD [1, 4, 11, 14, 26–28]. Early studies indicated a lower prevalence of CMHs, such as a 2004 study from the Framingham Study Offspring Cohort which found a 4.7% prevalence in 472 subjects [29]. However, more recent studies employing advanced imaging techniques and refined detection criteria have reported a higher prevalence of CMHs, leading to the current consensus based on pathological findings that approximately 50% of the older general population may have these microbleeds [2, 13, 30–32]. Additionally, in populations with significant vascular risk factors, the prevalence of CMHs is observed to be even higher, underscoring the impact of vascular health on the occurrence of these cerebral lesions. To illustrate this evolving view on the significant prevalence of CMHs in older adults, some key studies are reviewed below, highlighting the extent and implications of this condition in aging populations.

An important research effort, which involved 1965 participants from the Framingham Original and Offspring cohorts, found CMHs in 8.8% of the study population [25]. In a research project published in 2008 involving 1062 participants from the population-based Rotterdam Scan Study with an average age of 69.6 years, CMHs were assessed using 1.5 T MRI scans [26]. The study found a high overall prevalence of CMHs, which notably increased with age, ranging from 17.8% in individuals aged 60–69 years to 38.3% in those over 80. Interestingly, carriers of the APOE epsilon 4 allele were significantly more likely to have strictly lobar CMHs compared to non-carriers. In contrast, traditional cardiovascular risk factors, as well as the presence of lacunar infarcts and WMHs, were associated with CMHs located in deep or infratentorial brain regions, but not with lobar CMHs. In the Multi-Ethnic Study of Atherosclerosis (MESA), a comprehensive examination of CMHs in a diverse population was conducted using 3 T MRI SWI sequences [33]. The study involved 1016 participants without a history of stroke. The prevalence of CMHs was found to be significantly age-dependent, with 20% of participants aged 60 to 64.9 years exhibiting CMHs, and the prevalence increasing to 45% in individuals aged 85 years and older. In terms of microbleed locations, deep microbleeds were more commonly associated with older age, hypertension, and higher body mass index. These findings highlight the variability in the reported CMH prevalence rates, reflecting differences in study populations and advancements in imaging techniques.

Hypertension-induced CSVD may predominantly lead to the formation of deep CMHs, whereas CAA is more likely to drive the development of lobar CMHs [34]. This distinction highlights the differing pathophysiological mechanisms and regional impacts of these conditions on the brain’s microvasculature.

### Association between CMHs, cognitive decline, VCID, and AD

A growing body of evidence has established a clear association between CMHs and cognitive decline. CMHs are increasingly recognized as markers of cerebrovascular pathology that can exacerbate or contribute to the cognitive decline in conditions like VCID and AD.

Previous research has identified a significant relationship between the presence of CMHs and executive dysfunction [35]. This association, independent of other cerebrovascular changes, was particularly pronounced in patients with CMHs located in the frontal region and basal ganglia [35]. In the Rotterdam Scan Study, an investigation of 3979 elderly individuals without dementia, a higher number of CMHs was found to correlate with lower scores on the Mini-Mental State Examination (MMSE) and poorer performance in information processing and motor speed [36]. Particularly, the presence of five or more CMHs was associated with worse performance across multiple cognitive domains, highlighting an independent role of CMHs in the development of cognitive impairment [36]. In a follow-up study with a mean duration of 4.8 years, a significant association between the presence of more than four CMHs and cognitive decline was found [37]. Lobar microbleeds were linked to deterioration in executive functions, information processing, and memory function, whereas CMHs located in other brain regions correlated with declines in information processing and motor speed [37]. The presence of CMHs emerged as a significant risk factor for the development of both Alzheimer’s dementia and VCID with an age, sex, and education-adjusted hazard ratio of 2.02 [37]. Other studies also reached similar conclusions [38–44]. Complementing these findings, the Radboud University Nijmegen Diffusion Tensor and Magnetic Resonance Cohort (RUN DMC) Study found CMHs in 10.4% of 500 nondemented elderly patients, which were significantly associated with global cognitive function, psychomotor speed, and attention [45]. These cognitive correlations, predominantly driven by CMHs located in frontal, temporal, and strictly deep regions, were observed independently of other CSVD-related lesions like WMHs and lacunar infarcts, highlighting the clinical relevance of CMHs in understanding vascular contributions to cognitive decline [45]. The Vascular Mild Cognitive Impairment (VMCI)-Tuscany study, focusing on CSVD patients with mild cognitive impairment, found that nearly one-third of participants had CMHs [46]. The total count of CMHs in these patients correlated with impairments in attention, executive functions, and fluency domains [46]. Similarly, the DNA-Lacunar-2 multicenter study identified a significant association between CMHs and cognitive dysfunction, particularly affecting executive function and processing speed [47]. Additionally, the Rotterdam Study found that CMHs in deep or infratentorial brain regions also increase the risk for depressive disorders [48]. These varied studies collectively highlight the multifaceted impact of CMHs on cognitive health, indicating their pivotal role in the pathogenesis of cognitive and mood disorders in aging populations.

### Clinical evidence linking atherosclerosis, CSVD, and CMHs

Clinical and epidemiological studies have provided insights into the interconnectedness of atherosclerosis, CSVD, and the development of CMHs [26, 28, 49–52]. Atherosclerotic vascular disease manifests variably across different vascular beds, leading to a range of clinical conditions including carotid artery stenosis, acute myocardial infarction in the coronary arteries, ischemic strokes due to intracerebral artery occlusion and peripheral arterial disease (PAD) in the limb [53]. In this section, we overview the evidence linking these atherosclerotic vascular diseases to CSVD and CMHs.

In the cardiovascular segment of the Malmo Diet and Cancer Study, a large-scale, prospective population-based study in Sweden involving 6103 participants, researchers identified a significant association between midlife carotid artery atherosclerosis and CSVD [50]. This link was established based on the presence of specific MRI imaging signs indicative of CSVD, which included CMHs, lacunar infarcts, and WMHs [50]. The findings of a recent systematic review and meta-analysis indicate a significant association between advanced carotid artery stenosis and the presence of CMHs [52]. The analysis revealed an odds ratio of 1.95, with a 95% confidence interval ranging from 1.13 to 3.36, supporting the potential link between large artery atherosclerosis and the development of CSVD. Other studies also reached similar conclusions [54–56], confirming that advanced systemic atherosclerosis associates with various imaging markers of CSVD. In patients who have experienced ischemic stroke, CMHs are also closely linked to carotid artery atherosclerosis [57]. However, the aforementioned studies found no consistent correlation between atherosclerosis and the subsequent development of AD dementia or AD pathology [50].

In a study by Kim et al. involving 312 individuals aged 65 and older, a significant association was observed between increased coronary artery calcification scores and indicators of CSVD [51]. The study specifically noted strong associations between moderate-to-extensive coronary artery calcification and various manifestations of CSVD, such as CMHs (with an adjusted odds ratio and 95% confidence interval of 6.07 and 1.54–23.94), WMHs (4.99 and 1.33–18.73), and lacunar infarcts (5.04 and 1.86–13.63) [51]. Consistent with these observations, a cross-sectional analysis of data from the Age, Gene, Environment Susceptibility (AGES)-Reykjavik Study cohort of older adults showed that subjects with higher coronary artery calcification are more likely to have dementia, lower cognitive scores, and more CMHs and WMHs [58]. Other studies also reached similar conclusions [59–61]. In an investigation involving 782 high-risk individuals who were first-degree relatives of patients with early-onset coronary artery disease enrolled in the Genetic Study of Atherosclerosis Risk (GeneSTAR), a direct association was identified between the presence and volume of coronary artery plaques and volumes of WMHs [62]. An analysis of imaging markers of CSVD in participants of the Atherosclerosis Risk in Communities (ARIC) study, a community-based cohort investigation, demonstrated a higher prevalence of CMHs in individuals with a family history of coronary artery disease [63]. These findings not only suggest the existence of shared pathophysiological mechanisms across different vascular beds but also hint at the potential influence of common lifestyle risk factors or genetic predispositions for accelerated vascular aging within families.

Ischemic stroke and CSVD have a closely interlinked relationship where CSVD can be both a contributing factor to the occurrence of ischemic stroke and an indicator of heightened vascular risk, often associated with the atherosclerotic processes underlying ischemic strokes. Accordingly, in the Rotterdam Study, a comprehensive longitudinal population-based analysis, the presence, number, and location of CMHs were found to be linked with an increased risk of stroke among 4759 participants aged 45 years and older, over a follow-up period of approximately 5 years [64]. The study highlighted that the presence of CMHs was associated with a twofold increased risk of all types of strokes [64]. Notably, the risk escalated with an increasing count of CMHs [64]. Particularly, participants with CMHs located in areas indicative of CAA faced a fivefold increased risk of intracerebral hemorrhage [64]. Meanwhile, CMHs in other locations were associated with an elevated risk of both ischemic stroke and intracerebral hemorrhage [64]. These findings underscore the critical role of CMHs as a marker for increased stroke risk, particularly highlighting their significance in predicting the type of stroke based on their location within the brain.

Other studies demonstrated that patients who have experienced an ischemic stroke or transient ischemic attack (TIA) are at an increased risk of developing new CMHs [65, 66]. In older adults who experience acute ischemic stroke, CMHs [67] and other signs of CSVD are prevalent [68]. Notably, a high cardio-ankle vascular index — an indicator of systemic atherosclerotic burden — was independently linked to the presence of CMHs in these patients [67]. This association underscores the potential synergy between systemic atherosclerosis and the development of CMHs in the context of acute cerebrovascular events as well [67]. CMHs are also recognized as a significant risk factor for future intracerebral hemorrhage in patients who have experienced ischemic stroke [69–71]. Their presence indicates a heightened vulnerability within the entire cerebral vasculature, which can predispose these patients to subsequent larger hemorrhagic events. The presence of CMHs holds significant prognostic relevance for the long-term cognitive outcomes in stroke patients [72–80]. These relationships highlight the importance of monitoring CMHs and managing CSVD in the context of stroke care and prevention.

PAD, a common manifestation of systemic atherosclerosis in the limb vessels, is garnering attention for its potential link to cognitive impairment [81–86]. There is emerging evidence suggesting that individuals with PAD face an elevated risk of developing CSVD [87] and CMHs [27]. In older patients with chronic kidney disease, who are already at an increased risk for atherosclerotic vascular conditions including PAD, there is also a higher prevalence of CMHs [88]. However, this area of study requires further exploration to solidify these associations.

The aforementioned studies suggest that the risk factors and pathophysiological processes associated with atherosclerosis, such as endothelial dysfunction and chronic inflammation, may also play a role in the development of CSVD and subsequent formation of CMHs [89–92]. The common thread linking these conditions is the underlying age-related vascular pathology, which manifests with different clinical syndromes depending on the location of the diseased vessels in the body and across the spectrum from large vessels disease to microvascular changes.

#### Imaging signs of small vessel pathology in the retinal vasculature of patients with atherosclerotic diseases: insights from fundus imaging and optical coherence tomography

To comprehensively understand microvascular pathologies associated with atherosclerotic vascular diseases, it is crucial to directly study the structural and functional changes in the central nervous system’s microcirculation. Yet, limitations in in vivo brain vascular imaging make this challenging. Here, the retinal microvasculature offers a unique window, mirroring the cerebral circulation in anatomy, physiology and pathophysiology [93–131]. This similarity allows retinal examinations to serve as non-invasive tools for investigating CNS microvascular pathologies associated with systemic diseases.

Retinal imaging has evolved rapidly over the past few decades, allowing easy and non-invasive visualization of the retinal vasculature and neuronal structure [129, 130, 132]. Retinal fundus photography, a crucial tool for retinal disease assessment, captures color images of the retina. Additional detailed analysis of retinal vasculature characteristics, such as fractal dimension, tortuosity, and vessel caliber, can be performed using computer-assisted analysis programs like SIVA (Singapore I Vessel Assessment), VAMPIRE, ARIA, and IVAN [133]. Optical coherence tomography angiography (OCTA), a cutting-edge non-invasive imaging technique, distinctly visualizes various retinal and choroidal vascular layers without the need for dye injection [132]. This method not only yields detailed visualizations of the retinal vasculature but also provides extensive data on retinal blood flow, including measurements of foveal avascular zone area and capillary density. These capabilities enable more objective and accurate interpretation of retinal microcirculation images. OCTA’s ability to detect subtle changes in retinal microcirculation that correlate with intracranial blood flow makes it particularly valuable for assessing cerebral circulation in patients with atherosclerosis [132, 134–136]. Studies utilizing these methods have reported significant changes in retinal microvascular parameters associated with systemic pathologies, including large vessel atherosclerotic diseases [93–96, 98, 100–106, 109–119, 121, 123, 124, 126–128, 137, 138]. For example, internal carotid artery stenosis is associated with decreased retinal blood flow and reduced retinal microvascular density [134–136]. Additionally, significant correlations have been observed between impaired cerebrovascular reactivity and reduced retinal vessel density in these patients, suggesting parallel atherosclerosis-associated changes in both the retinal and the cerebral microcirculations [134–136]. Recent studies also report significant association between retinal microvascular parameters and coronary artery disease [96, 98, 138]. Overall, these insights indicate that retinal vascular parameters can serve as effective biomarkers for the early detection and monitoring of microvascular complications in atherosclerotic diseases. Continued research with longer-term follow-ups and larger sample sizes is necessary to further validate these findings and enhance our understanding of the relationship between retinal and cerebral microvascular health in the context of systemic atherosclerosis.

## Atherosclerosis as an age-related disease: pathophysiology and impact on large arteries

Atherosclerosis, primarily an age-related disease [139–150], is characterized by the progressive buildup of plaques within the arterial walls. These plaques, composed of lipids, cholesterol, calcium, and cellular debris, lead to the thickening and hardening of arteries, significantly impacting blood flow. The progression of atherosclerosis involves the gradual narrowing of arterial lumens. The disease typically progresses silently over decades, affecting the aorta and various arterial systems, including those of the brain, heart, extremities, gastrointestinal tract, and kidneys. It becomes clinically significant when it restricts the blood supply to essential organs. A critical juncture occurs when a thrombus forms on a ruptured atherosclerotic plaque, leading to complete blockage of blood flow and potentially resulting in severe ischemic events like heart attacks and strokes.

Atherogenesis is a complex process beginning with endothelial dysfunction and injury [151–156]. This initial step triggers a cascade of events, including the infiltration of low-density lipoprotein (LDL) cholesterol into the arterial wall and its oxidation. Oxidized LDL, a potent inflammatory agent, attracts immune cells and fosters the formation of fatty streaks, the forerunners of atherosclerotic plaques. The role of immune cells, particularly Clonal Hematopoiesis of Indeterminate Potential (CHIP), is critical in this context [145, 157–162]. CHIP, characterized by the expansion of blood cell clones with certain somatic mutations, are increasingly recognized for their contribution to chronic inflammation and atherosclerosis [159–162].

Atherosclerosis shows a preferential development in certain locations of large arteries, influenced by hemodynamic factors. It is a disease of high-pressure arterial systems; unlike veins, which are exposed to the same circulating factors but operate under lower pressure, only arteries are prone to develop atherosclerosis. This disease propensity is linked to areas of high wall tension, typically found in larger arteries under high intraluminal pressure. In contrast, smaller arteries and arterioles, with lower wall tension due to their smaller diameter and reduced pressure, are generally spared, despite being exposed to the same circulating factors.

Shear stress, the frictional force exerted by blood flow on the vessel wall, plays a pivotal role in atherosclerosis [163–165]. High shear stress, typically found in areas of unidirectional flow, exerts a protective effect on endothelial cells, stimulating the production of anti-inflammatory and anti-atherogenic factors like NO. This protective mechanism is one reason why exercise, which increases blood flow and shear stress, is beneficial in preventing atherosclerosis. Regular physical activity enhances endothelial function and reduces the risk of plaque formation. Interestingly, resistance arteries also experience higher shear stress compared to large arteries, contributing to their relative resistance to atherogenesis. Despite being exposed to the same circulating risk factors as larger arteries, the heightened shear stress in these smaller vessels, together with the lower wall tension, aids in maintaining endothelial integrity and preventing plaque formation.

Epidemiological and pathological studies have consistently shown that atherosclerotic plaques tend to form preferentially at bifurcations and branching points [165]. The predilection of atherosclerosis for arterial bifurcations and branching points is a result of the complex interplay between altered hemodynamics, endothelial response to shear stress, concentration of mechanical stress, and the subsequent localization of atherosclerotic changes at these sites. These factors collectively contribute to making these vascular regions hotspots for the development of atherosclerotic plaques. Arterial bifurcations and branching points are regions where blood flow patterns are disrupted. Instead of unidirectional flow observed in straight arterial segments, these points experience complex flow patterns, including turbulent and oscillatory flow. This altered hemodynamics leads to variations in shear stress, with certain areas experiencing lower shear stress than others. Bifurcations and branching points are also areas where mechanical stress is concentrated. The physical forces acting on the vessel walls at these points are higher due to the geometry of the vessels and the nature of blood flow.

Aging is a crucial pathogenic factor in atherosclerosis. It is a quintessential age-related disease and there is a growing literature demonstrating that the “pillars of aging”, a concept encompassing various fundamental biological processes that contribute to aging, play a significant role in the pathogenesis of atherosclerosis [23, 24, 140, 142, 145, 147, 149, 166–168]. These pillars, or “hallmarks of aging”, provide a framework to understand how aging at the cellular and molecular level influences the development of age-related diseases, including atherosclerosis.

Cellular oxidative stress is a hallmark of aging and it plays a critical role in the pathogenesis of atherosclerosis as well [23, 24, 169]. It acts as a pivotal mediator between risk factors and atherogenesis, influencing multiple stages of the disease from endothelial dysfunction and LDL oxidation to inflammation and plaque stability [169]. In endothelial cells, increased levels of reactive oxygen species (ROS) reduce the bioavailability of nitric oxide (NO) and activate pro-inflammatory signaling pathways, including NF-kB, which mediate endothelial activation. Moreover, oxidative stress plays a significant role in LDL oxidation, a process central to the development of atherosclerotic plaques. Oxidized LDL (oxLDL) is more atherogenic than native LDL as it is readily taken up by macrophages, leading to foam cell formation — one of the earliest cellular changes in atheroma development. OxLDL also promotes further release of ROS and inflammatory cytokines, creating a vicious cycle of inflammation and oxidative damage. Additionally, oxidative stress can contribute to vascular smooth muscle cell proliferation and migration, processes that are involved in the remodeling of the arterial wall and plaque instability. DNA damage induced by ROS also plays a role in genomic instability and the senescence of vascular cells, further aggravating the atherosclerotic process.

Genomic instability, characterized by DNA damage and mutations, increases with age [147, 149, 170–173]. In the context of atherosclerosis, such genomic alterations can affect vascular cells, leading to dysfunctional cellular responses and contributing to the development of vascular lesions. Accumulated DNA damage in endothelial and smooth muscle cells can disrupt normal cell function and promote inflammatory responses, both key factors in atherogenesis. Additionally, CHIP, a phenomenon where certain blood cell clones with specific somatic mutations expand, is increasingly recognized as a contributor to this process [145, 157–162]. CHIP is associated with heightened systemic inflammation and the presence of mutated white blood cell clones in atherosclerotic plaques, further underscoring the role of age-related genomic instability in vascular health.

With aging, an increased number of cells enter a state of senescence, often as a result of accumulated oxidative stress-induced DNA damage [148, 149, 174–180]. This increase in senescent cells has been notably observed within atherosclerotic plaques. Senescent cells in the vascular system can secrete a variety of pro-inflammatory and matrix-degrading molecules, known as the senescence-associated secretory phenotype (SASP) [23, 24]. This can promote chronic inflammation and plaque instability in atherosclerosis. Shortened telomeres in vascular cells can also lead to cellular senescence, contributing to plaque formation and progression.

Aging is associated with a decline in the ability of cells to maintain protein homeostasis, or proteostasis. In atherosclerosis, impaired proteostasis can result in the accumulation of dysfunctional proteins within vascular cells, contributing to cellular stress, inflammation, and ultimately, the destabilization of atherosclerotic plaques [23].

Changes in the epigenetic landscape, such as DNA methylation patterns, histone modifications, and non-coding RNA expression, are hallmarks of aging. In atherosclerosis, epigenetic modifications can influence the expression of genes involved in lipid metabolism, inflammation, and endothelial function [168, 181–183]. These changes can alter the behavior of vascular cells and the immune cells within plaques, driving the atherosclerotic process.

Nutrient-sensing pathways, which include mTOR, AMPK, and sirtuins, play critical roles in metabolic regulation and have been linked to aging and longevity. In atherosclerosis, alterations in these pathways can influence lipid metabolism, inflammation, and endothelial function, thereby contributing to plaque development and vulnerability [166, 180, 184–189].

Mitochondria, the cellular powerhouses, become less efficient with age. Mitochondrial dysfunction in vascular cells can lead to increased oxidative stress and reduced bioenergetic capacity, both of which are implicated in endothelial dysfunction and the inflammatory processes central to atherogenesis [141, 147, 171, 190–192]. Mitochondrial dysfunction leads to a decrease in ATP production through oxidative phosphorylation, a process vital for maintaining cellular energy balance in endothelial cells [193–195]. Furthermore, mitochondrial dysfunction affects the glycolytic pathway, an alternative source of cellular energy [193, 196]. As mitochondria become less efficient, they produce fewer ATP molecules and generate increased levels of ROS, contributing to oxidative stress within the aging vascular system [197–202]. Mitochondria-derived ROS are key factors in endothelial dysfunction and vascular inflammation, that contribute to the development of atherosclerosis and CSVD [199, 201, 203–205].

Vascular risk factors known to accelerate cellular and molecular aging processes, thereby exacerbating atherogenesis in larger arteries, include hypertension, high cholesterol levels, smoking, diabetes mellitus, obesity, consumption of unhealthy diets [206], and a sedentary lifestyle. Each of these risk factors can accelerate one or more of the cellular mechanisms of aging, such as oxidative stress, inflammation, or cellular senescence, which in turn contribute to the development and progression of atherosclerosis. For instance, hypertension can induce oxidative stress and endothelial dysfunction, while high cholesterol levels facilitate the formation and oxidation of LDL, accelerating endothelial damage and inflammation. Smoking is known to cause systemic oxidative stress, DNA damage and exacerbate inflammatory responses, further promoting vascular aging. Obesity, consumption of an unhealthy diet, and lack of physical activity can lead to metabolic disturbances, mitochondrial dysfunction and oxidative stress, cellular senescence and inflammation, all of which are conducive to atherogenesis. Genetic predispositions also significantly influence the disease’s risk and severity, often by modulating these same cellular aging processes.

## The continuum of vascular aging: linking atherosclerosis to CSVD

Vascular aging is a progressive, multifactorial process impacting the entire vascular tree, from large arteries to the microvasculature [23, 24]. This concept posits that vascular aging should not be perceived as a series of isolated events in different vessel sizes but rather as a continuous spectrum of interconnected changes throughout the vascular network. Within this spectrum, the pathological alterations observed in larger vessels, such as those occurring in atherosclerosis, are fundamentally connected to the changes in the microvasculature, contributing to the development of CSVD. Similarly, vascular risk factors known to expedite aging and atherogenesis in larger arteries are also implicated in accelerating microvascular aging, thereby promoting the development and progression of CSVD. This interrelation underscores a comprehensive approach to understanding vascular health, spanning from macroscopic arterial changes to microvascular alterations.

In this section, we will first delve into the shared cell-autonomous mechanisms of aging that contribute to both atherogenesis and CSVD. Subsequently, we will explore the role of non-cell-autonomous mechanisms of aging, including the impact of endocrine factors, in driving cellular aging processes that affect both large arteries and the cerebral microcirculation simultaneously. This dual perspective offers a more complete understanding of the aging processes in the vascular system, highlighting how systemic factors and local cellular changes coalesce to influence vascular health and disease across the entire vascular tree.

### Shared mechanisms of aging linking atherosclerosis to microvascular pathology

The connection between atherosclerosis and CSVD can be understood through several key shared mechanisms of aging [22–24, 86, 199–201, 207–223]. Akin to the changes observed in large vessels, the aging process in the microcirculation is marked by a notable increase in cellular production of ROS [20, 199, 200, 223–227]. Critical mechanisms driving this include age-related cellular NAD^+^ depletion, SIRT1 dysregulation, and the resultant rise in mitochondrial ROS generation [199, 223]. Increased microvascular oxidative stress contributes to endothelial dysfunction [199, 223], a key feature of CSVD, by impairing nitric oxide availability and promoting inflammatory processes [89–92]. Endothelial activation results in the expression of adhesion molecules and the recruitment of inflammatory cells and an increased propensity for thrombosis. These alterations in the microvasculature are intricately linked to several key pathological features of CSVD. Specifically, oxidative stress and endothelial dysfunction are associated with the disruption of the blood brain barrier (BBB) [228, 229], impairment of neurovascular coupling responses [199, 223, 225], and the genesis of lacunar infarcts and CMHs [20].

Accumulated DNA damage, largely driven by oxidative stress, also triggers cellular senescence within the microvasculature [215, 216, 230]. This process is particularly evident in endothelial cells, where senescence contributes significantly to microvascular pathologies. Senescent cerebromicrovascular endothelial cells, exhibiting the SASP, secrete a range of pro-inflammatory cytokines and matrix-degrading enzymes [231]. This cascade of molecular events exacerbates microvascular damage and plays a substantial role in the development of CSVD features [22, 213, 215, 216, 232]. Recent research has provided promising insights into the rejuvenation of the cerebral microcirculation through the targeted removal of senescent cells [22, 213, 215, 216]. Studies utilizing pharmacological or genetic interventions to deplete senescent cells in mouse models of aging have shown remarkable results, including the restoration of endothelial function, improvements in NVC responses, and enhanced BBB integrity [22, 213, 215, 216]. Notably, the use of the BCL-2 inhibitor senolytic drug Navitoclax has been shown to mitigate the development of CMHs induced by hypertension in aged mice [22]. This finding is particularly significant, as it suggests a potential therapeutic approach for preventing age-related microvascular changes that contribute to the pathogenesis of CSVD.

### Cell non-autonomous mechanisms of aging driving both atherosclerosis and microvascular pathologies

Cell non-autonomous mechanisms of aging refer to external factors and systemic influences that drive aging processes in both atherosclerosis and microvascular pathologies. These mechanisms often involve complex interactions between different cell types, tissues, and organ systems, highlighting the systemic nature of vascular aging.

#### Chronic systemic low-grade inflammation

Chronic systemic low-grade inflammation, commonly referred to as “inflammaging”, is a hallmark of the aging process [23, 24, 233, 234]. It is a key cell non-autonomous factor that exacerbates both atherosclerosis and microvascular pathologies [23, 24, 207]. This systemic inflammation can arise from various sources, including accumulation of senescent cells [22, 143, 174, 175, 177, 213, 215, 216, 230, 232], activation of innate immune system [235] and dysfunction and heightened inflammatory status of adipose tissue [236–246]. Senescent cells, which accumulate with age in adipose tissue and other organs, secrete pro-inflammatory cytokines, chemokines, and growth factors, known as the SASP, heightening the systemic inflammatory status [143, 175, 177, 230–232, 247]. As individuals age, the immune system also undergoes changes that predispose it to a pro-inflammatory state, contributing to systemic inflammation [248–250]. These inflammatory mediators affect vascular cells, promoting atherosclerotic plaque formation and microvascular damage characteristic of CSVD. In the context of atherosclerosis, chronic low-grade inflammation contributes to the initiation and progression of the disease. Inflammatory cytokines can promote endothelial dysfunction, a critical early step in atherogenesis. They also facilitate the recruitment, migration and activation of immune cells within the arterial wall, contributing to plaque formation, growth, and destabilization. In CSVD, inflammaging can lead to endothelial dysfunction, blood–brain barrier disruption, and increased vascular permeability [89–92]. The chronic inflammatory milieu is conducive to microvascular damage, which is manifested in various CSVD features like WMHs, lacunar infarcts, and CMHs.

#### Endocrine factors vascular aging: the role of age-related IGF-1 deficiency

Endocrine factors, particularly hormonal changes associated with aging, play a significant role in vascular health [202, 214, 218, 219, 251]. One of the key age-related hormonal changes in humans is the significant decline in circulating levels of insulin-like growth factor 1 (IGF-1) [219]. IGF-1 is crucial for maintaining vascular homeostasis, and its deficiency or altered signaling with age has profound effects on both atherogenesis [140, 252–259] and microvascular pathologies [15, 18, 214, 219, 220, 260–268].

IGF-1 has protective effects on the endothelium [214, 253, 260, 266, 269–273]. Age-related declines in IGF-1 contribute to endothelial dysfunction [266], an early and pivotal event in atherogenesis. Reduced IGF-1 signaling impairs the production of NO [266], attenuates vascular oxidative stress resilience [274], and exacerbates cellular and mitochondrial oxidative stress [256, 275]. IGF-1 has anti-inflammatory properties [253, 256, 258, 276]. Lower levels of IGF-1 can lead to a pro-inflammatory state in the vascular system, thereby accelerating the development and progression of atherosclerotic plaques. In preclinical porcine models, IGF-1 deficiency exacerbates atherogenesis [253, 256, 258, 276].

IGF-1 imparts a range of protective effects on cerebromicrovascular health, playing a pivotal role in mitigating the vascular changes associated with aging [15, 18, 214, 219, 220, 260, 263, 264, 266–268]. In preclinical studies, IGF-1 deficiency has been shown to induce microvascular rarefaction and reduce cerebral blood flow, thus mirroring the vascular alterations seen in aging [18, 268]. The influence of IGF-1 extends to both pro-angiogenic, anti-apoptotic and anti-senescence actions on cerebromicrovascular endothelial cells, underscoring its multifaceted role in maintenance of the cerebral microcirculatory network [214, 277–280]. Additionally, IGF-1 plays a crucial role in counteracting the functional effects of aging on the cerebral microcirculation, effectively preserving its youthful functionality [210, 219, 220]. This is particularly evident in older adults, where lower levels of IGF-1 correlate with impaired NVC responses [220]. Experimental models in mice have reinforced this connection, demonstrating that induced IGF-1 deficiency leads to significant impairments in NVC, mimicking the aging phenotype [266]. Additionally, genetic disruptions in IGF-1 signaling, such as the knockdown of the IGF1R, have been shown to impair both endothelial and astrocytic components of NVC responses, highlighting the critical role of IGF-1 in this process [260, 261]. The contribution of IGF-1 signaling to vascular integrity is further emphasized in its role in maintaining the BBB [214]. Pertinent to this review, murine models that are either deficient in IGF-1 or demonstrate disrupted IGF-1 signaling specifically in the microvasculature, display a marked increase in microvascular fragility [15, 18]. This heightened vulnerability is directly linked to an increased likelihood of developing CMHs [15, 18]. Detailed analyses of these IGF-1 deficient models reveal pathological remodeling of the vascular wall, leading to compromised structural integrity [263]. The biomechanical and transcriptomic alterations in the vascular wall due to IGF-1 deficiency [18, 263] underscore its importance in maintaining cerebromicrovascular health and stability. In summary, IGF-1 emerges as a key factor in preserving cerebromicrovascular integrity and function. Its deficiency leads to a spectrum of microvascular impairments that mirror aging-related changes, highlighting the potential of targeting IGF-1 pathways in addressing age-associated cerebromicrovascular pathologies.

#### Alteration in the gut microbiome

The gut microbiome, a complex and dynamic ecosystem of microorganisms residing in the gastrointestinal tract, undergoes significant changes as part of the aging process [281–284]. With age, the diversity and balance of the gut microbiota can shift, often leading to a state known as dysbiosis [281–285]. This imbalance is characterized by a reduction in beneficial microbes and an increase in pathogenic ones. Such changes can disrupt the gut barrier function, leading to increased intestinal permeability, commonly referred to as “leaky gut” [282, 286–288].

Dysbiosis in the aging gut is linked to the increased translocation of bacterial products into the systemic circulation. This can trigger chronic low-grade inflammation, a key contributor to inflammaging [282, 286–288]. The gut microbiome also plays a crucial role in modulating the immune system. Changes in microbiota composition can affect immune cell maturation and response, potentially leading to altered inflammatory and immune responses [289–291]. These alterations can have far-reaching effects beyond the gut, influencing systemic inflammation, immune function, and, by extension, vascular health.

Emerging evidence suggests that the gut microbiome may also influence microvascular health (“gut-brain axis”), potentially impacting the pathogenesis of CSVD. This may occur through mechanisms such as increased systemic inflammation and direct effects on cerebral microvessels mediated by gut-derived metabolites and inflammatory mediators [292–296].

## Microvascular aging and CMHs

Microvascular aging significantly contributes to the pathogenesis of CMHs [20, 297, 298]. Recent studies have identified specific cellular and molecular aging processes that compromise the structural integrity of cerebral microvessels [20, 22]. Here, we explore key mechanisms, primarily based on preclinical studies, that link microvascular aging to the development of CMHs.

There is growing preclinical evidence that age-related cellular oxidative stress plays a critical role in promoting CMHs [20]. Increased production of ROS within the microvasculature can lead to the activation of matrix metalloproteinases (MMPs), enzymes that degrade extracellular matrix components and weaken the vessel walls [20]. Preclinical studies have demonstrated that antioxidative treatments can prevent the formation of CMHs, supporting the hypothesis that mitigating oxidative stress is key to preserving microvascular integrity [20]. The primary mechanism appears to be the reduction of ROS-mediated MMP activation, which otherwise contributes to vascular fragility [20].

There is also evidence highlighting a critical role for age-related endocrine changes in the genesis of CMHs. In particular, IGF-1 deficient mice exhibit an increased propensity to develop CMHs, mirroring the effects of aging on the microvasculature [18]. The likely mechanisms involve increased ROS-mediated MMP activation and pathological vessel remodeling, characterized by media atrophy, altered synthesis of extracellular matrix (ECM) components and impaired structural adaptation to hypertension [18, 263].

The increased presence of senescent cells in the aging microcirculation is another factor contributing to CMHs [22]. Senescent cells, through the SASP, release various factors and matrix-degrading enzymes, including MMPs [299], that contribute to the remodeling of the extracellular matrix and promote microvascular fragility [23, 24]. Senescent cells may also hinder the structural and functional adaptation of cerebral vessels to hypertension [298]. Normally, cerebral resistance arterioles adapt to hypertension through structural remodeling, such as media hypertrophy, to prevent microvascular damage [300]. Senescence might disrupt these adaptive processes, leading to increased microvascular fragility. Furthermore, SASP factors can induce paracrine senescence, spreading senescent effects to neighboring cells and amplifying the impact on microvascular function [23, 24]. Preclinical studies have shown that senolytic treatments, which target and eliminate senescent cells, can attenuate the formation of CMHs in aged mice [22]. This finding underscores the potential therapeutic value of targeting cellular senescence to mitigate microvascular aging and reduce the risk of CMHs. Of note, increased endothelial senescence in the cerebral microcirculation has been also linked to BBB disruption and neurovascular dysfunction [216, 301]. Accelerated microvascular aging in a range of pathological conditions, characterized by enhanced oxidative stress, increased cellular senescence, and pathological microvascular ECM remodeling, likely exacerbates the formation of CMHs. As the microvasculature ages, these processes intensify, leading to increased vascular fragility and susceptibility to rupture.

In conclusion, microvascular aging processes, including oxidative stress, impaired IGF-1 input, and cellular senescence, play a significant role in the development of CMHs. Tackling these underlying mechanisms may offer new avenues for preventing and treating CMHs, a common manifestation of aging in the cerebral microcirculation.

## Exacerbating factors: hypertension and amyloid pathology

Hypertension and amyloid pathology are significant exacerbating factors in the progression of CSVD and the formation of CMHs [14, 17, 221]. Their impact is multifaceted, accelerating the microvascular aging processes.

Chronic hypertension per se can lead to endothelial dysfunction in the cerebral microvasculature [302–306]. It upregulates both NADPH oxidase and mitochondria-dependent ROS production, decreasing NO bioavailability, activating inflammatory processes, and causing structural damage to endothelial cells [298, 302–307]. Sustained high blood pressure can also compromise the integrity of the BBB [298]. The continuous stress and strain on cerebral vessels due to high blood pressure increase the propensity for vessel rupture, directly contributing to the formation of CMHs.

Amyloid-beta (Aβ) deposits in the walls of cerebral microvessels weaken their structural integrity. This process, known as cerebral amyloid angiopathy (CAA), is a significant factor in the development of CMHs [308, 309]. CAA can disrupt normal vascular function, affecting vasoreactivity and leading to further damage and instability in the microvasculature [310–312]. Studies in preclinical models demonstrate that the presence of amyloid pathology can amplify the detrimental effects of hypertension on cerebral vessels, increasing the likelihood of vessel rupture and CMH formation [17].

Both hypertension and amyloid pathology can accelerate the microvascular aging processes, hastening the decline in vascular function and health. This accelerated aging process involves increased oxidative stress, inflammation, and endothelial dysfunction [302, 313–319], all of which contribute to the progression of CSVD and the increased incidence of CMHs. By understanding the combined impact of hypertension and amyloid pathology on microvascular aging, better strategies can be developed to prevent or mitigate the damaging effects of these factors on cerebral vasculature.

## Implications for clinical practice and future research

The recent advancements in understanding vascular aging and its association with both CSVD and atherosclerosis carry profound implications for clinical practice and future research [320]. This knowledge paves the way for more effective management strategies and the development of novel therapeutic approaches, crucial for preventing both cardiovascular incidents and cognitive decline. The recognition of shared mechanisms in atherosclerosis and CSVD suggests the need for an integrated approach in managing these conditions. Clinical treatments might need to focus not just on symptom management but also on addressing the underlying vascular aging processes. Effective control of vascular risk factors, such as hypertension, diabetes mellitus, and hyperlipidemia, becomes even more crucial. Lifestyle interventions and pharmacological treatments targeting these risk factors could be beneficial in slowing down vascular aging and preventing complications, including cognitive impairment. Advances in understanding vascular aging may also lead to the identification of early biomarkers, facilitating timely detection and management of CSVD, particularly in patients with atherosclerosis.

**Fig. 1:**
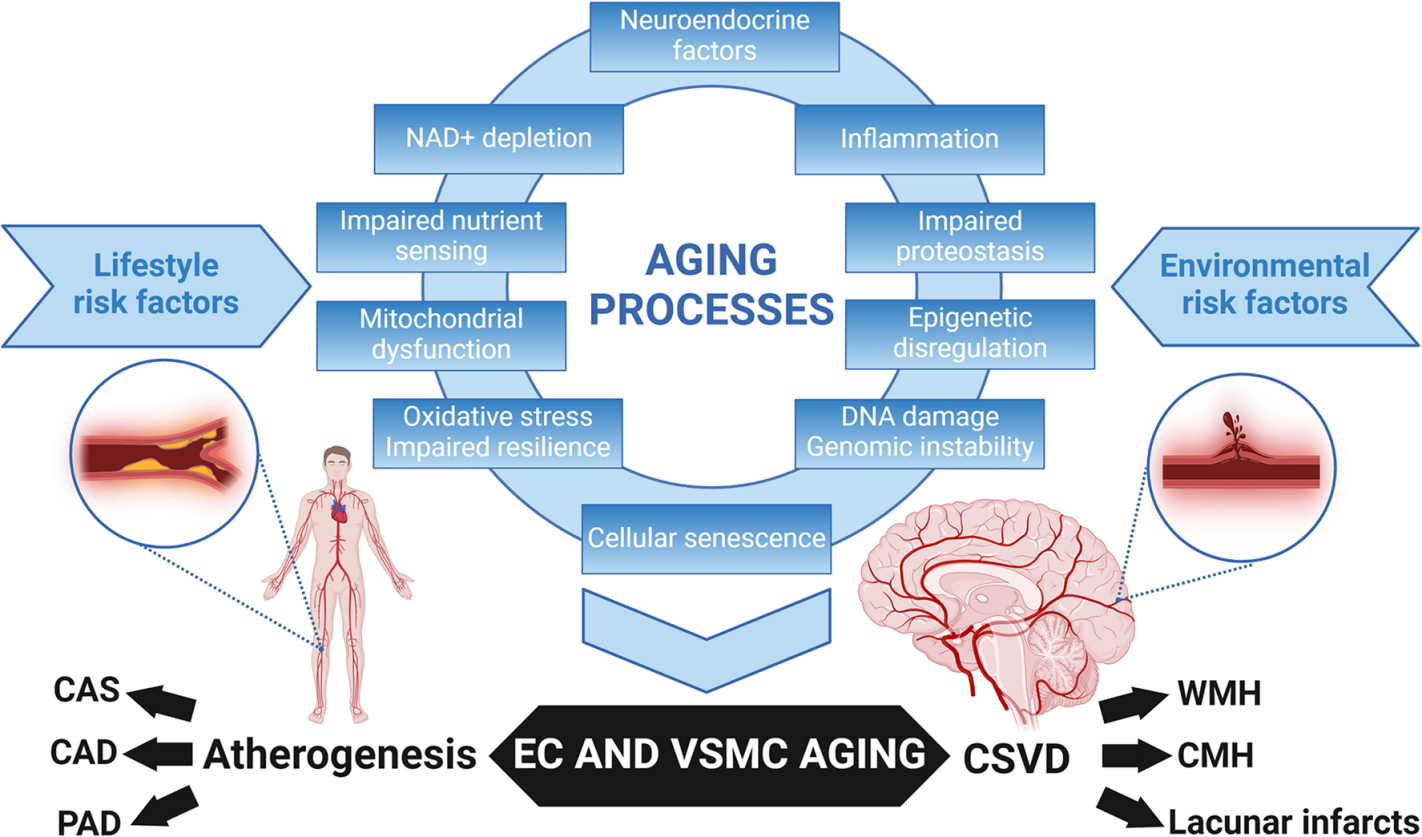
Continuum of accelerated vascular aging: bridging atherosclerotic diseases and cerebral small vessel disease (CSVD). This schematic figure illustrates the central role of fundamental cellular and molecular mechanisms of aging, collectively driving the progression of both macrovascular and microvascular aging. The top portion of the figure highlights the interconnected hallmarks of aging, including oxidative stress, mitochondrial dysfunction, cellular senescence, and a heightened inflammatory state. These synergistic aging processes induce age-related functional and phenotypic changes in endothelial cells (EC) and vascular smooth muscle cells (VSMC), contributing to the pathogenesis of a range of vascular diseases associated with aging. Lifestyle risk factors such as unhealthy diets, smoking, sedentary behavior, and environmental risk factors like air pollution further accelerate these vascular aging processes. This leads to atherogenesis in large arteries, manifesting as carotid artery stenosis (CAS), coronary artery disease (CAD), and peripheral artery disease (PAD), as well as cerebral small vessel disease (CSVD) in the cerebral microcirculation. The model suggests that atherosclerotic vascular diseases and CSVD originate from common aging processes, accounting for the frequent co-occurrence of CAS, CAD and/or PAD, and neuroimaging manifestations of CSVD, such as cerebral microhemorrhages (CMHs) and white matter hyperintensities (WMH), in the elderly

With cellular senescence being a key contributor to vascular aging, senolytic drugs that selectively clear senescent cells offer a promising therapeutic approach. These could potentially reduce inflammaging and mitigate vascular damage in both CSVD and atherosclerosis. Given the role of oxidative stress in vascular aging, novel, targeted antioxidative therapies could be explored for their potential in protecting against microvascular damage in high-risk patients. Investigating therapies that modulate endocrine factors influencing vascular aging could be beneficial in both prevention and treatment. Further translational research is needed to unravel the detailed mechanisms by which aging processes affect both large arteries and the cerebral microcirculation. Understanding these pathways at the molecular level could open new therapeutic avenues. There is also a need for long-term studies to understand the progression of vascular aging and its impact on CSVD and atherosclerosis over time. Research into individual variability in vascular aging could lead to more personalized approaches in treating and preventing CSVD in high-risk patients. By incorporating these insights into clinical practice and future research [320], there is potential to significantly improve the management of CSVD and enhance the quality of life for individuals affected by this condition Fig. 1.

## Conclusion

The exploration of vascular aging, particularly in the context of CSVD, highlights a critical aspect of brain health that has far-reaching implications. Understanding the continuum of vascular aging — from atherogenesis in large arteries to the age-related structural and functional alterations in the microvasculature — is essential for a comprehensive approach to diagnosing, preventing, and treating CSVD. This continuum perspective emphasizes that vascular aging is a systemic process that impacts the entire circulatory system. The interplay between large vessel diseases like atherosclerosis and microvascular pathologies such as CSVD underscores the need for a holistic approach in medical research and clinical practice. Geroscience discoveries are shedding light on novel mechanisms that may simultaneously promote atherogenesis and CSVD. Understanding these mechanisms could lead to innovative therapeutic strategies targeting the root causes of vascular aging, ultimately preventing both large vessel diseases and microvascular complications like CMHs. By recognizing the interconnected nature of vascular aging, clinicians and researchers can better identify risk factors, develop targeted therapies, and implement effective preventive measures. Furthermore, this understanding encourages a shift in focus from treating individual symptoms to addressing the underlying mechanisms of vascular aging. Such an approach could lead to more effective strategies for slowing or even reversing aspects of vascular aging, with the potential to significantly impact patient outcomes in both cardiovascular and cerebrovascular diseases.
